# Parieto-Occipital Alpha and Low-Beta EEG Power Reflect Sense of Agency

**DOI:** 10.3390/brainsci11060743

**Published:** 2021-06-03

**Authors:** Hani M. Bu-Omer, Akio Gofuku, Kenji Sato, Makoto Miyakoshi

**Affiliations:** 1 Graduate School of Interdisciplinary Science and Engineering in Health Systems, Okayama University, 3-1-1 Tsushimanaka, Kita-Ku, Okayama 700-8530, Japan; 2 Hadhramout Foundation of Invention and Advancement of Science, Mukalla, Hadhramout, Yemen; 3 Department of Anesthesiology and Intensive Care Medicine, Kawasaki Medical School, 577 Matsushima, Kurashiki, Okayama 701-0192, Japan; k.sato@med.kawasaki-m.ac.jp; 4 Swartz Center for Computational Neuroscience, Institute for Neural Computation, University of California San Diego, 9500 Gilman Drive, La Jolla, CA 92093-0559, USA; mmiyakoshi@ucsd.edu

**Keywords:** sense of agency, electroencephalography (EEG), mirror visual feedback, virtual reality, delayed visual feedback

## Abstract

The sense of agency (SoA) is part of psychophysiological modules related to the self. Disturbed SoA is found in several clinical conditions, hence understanding the neural correlates of the SoA is useful for the diagnosis and determining the proper treatment strategies. Although there are several neuroimaging studies on SoA, it is desirable to translate the knowledge to more accessible and inexpensive EEG-based biomarkers for the sake of applicability. However, SoA has not been widely investigated using EEG. To address this issue, we designed an EEG experiment on healthy adults (*n* = 15) to determine the sensitivity of EEG on the SoA paradigm using hand movement with parametrically delayed visual feedback. We calculated the power spectral density over the traditional EEG frequency bands for ten delay conditions relative to no delay condition. Independent component analysis and equivalent current dipole modeling were applied to address artifact rejection, volume conduction, and source localization to determine the effect of interest. The results revealed that the alpha and low-beta EEG power increased in the parieto-occipital regions in proportion to the reduced SoA reported by the subjects. We conclude that the parieto-occipital alpha and low-beta EEG power reflect the sense of agency.

## 1. Introduction

In recent years, research interest in two types of self-senses, sense of ownership (SoO) and sense of agency (SoA), has been increasing. The SoO is the sense one feels that one’s body parts, thoughts, or emotions belong to one. For example, when one’s hand is moved, one feels SoO irrespective of whether one initiated the movement voluntarily or the hand is moved by external force [1]. The SoA, on the other hand, refers to the sense of initiating and controlling actions to influence events in the outside environment. For example, when one moves a hand or think about something, one feels that s/he caused the hand moving or started the train of own thoughts. The SoO and SoA are considered psychophysiological modules that consists the sense of self, and an interruption in their consistency reported under some clinical conditions provides clues about the disorder and information useful for diagnosis and treatment.

Clinical studies on SoO and SoA investigated patients with chronic pain conditions including Complex Regional Pain Syndrome (CRPS), hemiplegia, and phantom limb [2,3,4,5,6] and reported altered SoO and SoA [7,8,9]. A study on CRPS reported a rather complicated interaction between pain and disturbing body senses in the affected parts, and proposed a treatment strategy that targets cortical areas to monitor and enhance body perception and pain symptoms in parallel [7]. A functional MRI (fMRI) study using virtual reality to investigate the neural correlate found mainly two brain regions responsible for the SoA: the prefrontal cortex including the cingulate cortex as a leading network processing a mismatch detection, and the inferior parietal lobule as a following network that generates the SoA [10]. An EEG study also reported modulation of the alpha-band EEG power (8–12 Hz) within the anterior frontal area reflecting the SoA [11]. Although MRI provides high spatial resolution, it is desirable to translate the MRI-based biomarkers to the EEG-based biomarkers for future application in terms of applicability. However, an EEG study replicating the result [11] is lacking. Finally, as is the common limitation in EEG studies, the spatial resolution of the reported results is limited, and the influence of artifacts from ocular and muscular potentials was not fully validated. These problems need to be addressed to advance the possibility of EEG-based biomarkers in the study of the SoA.

To replicate and extend our understanding on the sensitivity of EEG on the SoA paradigm, we designed an EEG experiment using healthy subjects. The goal of this study was to determine the EEG correlate of the SoA by using a behavioral task of hand movement with parametrically delayed visual feedback. To address the issue of volume conduction and artifact rejection, we used independent component (IC) analysis (ICA) and equivalent current dipole modeling to determine the locations of ICs of interest. Results showed that the spectral EEG activities in alpha and early-beta EEG frequency bands, mainly in parieto-occipital regions, are the main correlates of the SoA modulation.

## 2. Materials and Methods

In this work, we carried out a behavioral experiment to study both the implicit and explicit measures of the SoA. Electroencephalogram (EEG) recordings were obtained in the first part of the experiment to implicitly assess the neural correlate of the SoA fluctuations in response to disruption in the visual perception of individual’s body movement by introducing different amounts of time delay to the visual feedback of hand movement. The explicit SoA was assessed in this experiment by asking the participants to rate their perceived SoA after each time-delay condition applied to the video streaming of their real hand movement. The detailed description of the experiment design and tasks is presented in the following subsections.

### 2.1. Participants

Fifteen healthy volunteers have participated in the experiment. All of them are students at Okayama University (3 females, 12 males, age range 22–35 years, mean 28.5 ± 4.5 years). Prior to the day of the experiment, the participants were instructed to get enough sleep and wash their hair. We gave them explanations about the procedure of the experiment and answered their questions. All volunteers had normal or corrected to normal vision, and none of them had a history of neurological disorders.

### 2.2. Experimental Design and Procedure

The experiment consisted of two parts. In the first part, EEG data were recorded from the subjects while performing the hand movement task. When the first part was finished, the EEG electrode cap was removed, and participants washed their heads and took a 10-minute break. In the second part, the participants gave subjective ratings of the SoA after each task condition using a numeric scale.

**Figure 1:**
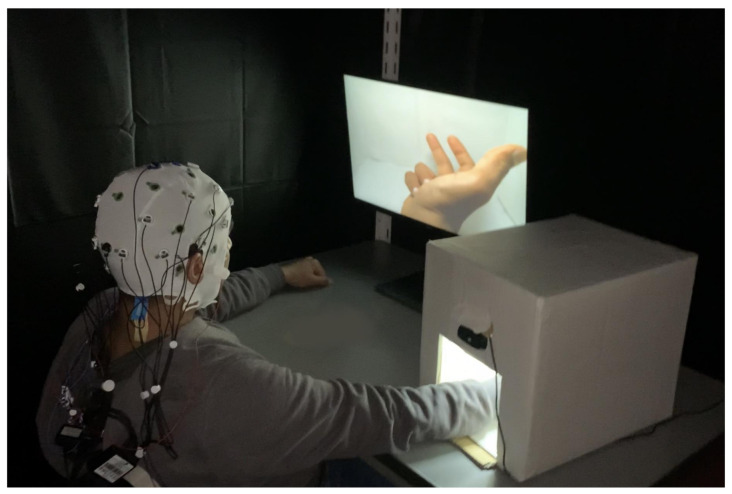
Environment of the EEG experiment. A participant performs the experiment’s tasks while monitoring his movement by watching the video stream on the front screen. The movement displayed on the screen can be delayed according to different time delay conditions.

The EEG experiment was performed in a chamber that was dimly lit and acoustically isolated. A small white box was designed to visually hide the actual participant’s hand movement (Figure 1). Inside the white box, a webcam was attached so that the hand movement was streamed on the display monitor in front of the subject. The tasks in both the first and the second parts were identical except that the subjects were instructed to report the perceived SoA in the second part.

**Figure 2:**
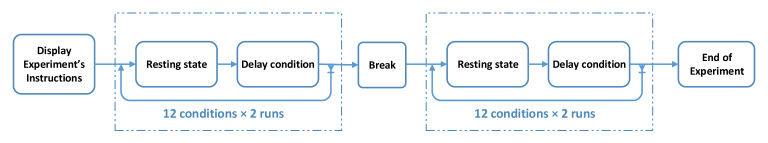
The block diagram of the experiment tasks and procedure. Each delay condition is displayed randomly within four condition cycles with a short break after the first two cycles.

During the experiment, the subjects were comfortably seated and placed their right hand inside the white box. They were asked to move the individual fingers sequentially while looking at the display monitor. The image of hand movement was displayed on the monitor with the parametrically controlled delay for each block-separated 20-s condition from 0 to 1000 ms with a 100 ms step. The subjects were instructed to maintain their pace and not follow the movement of the visual feedback. Note that small initial delays due to the electronic device were ignored in this experiment. In addition to the delay conditions, there was the “M” condition, in which the subjects moved their hand while watching a green screen. Before each of the delay conditions, there was a 20-s resting period, in which the subjects stopped hand movement and relaxed while watching a grey screen. Thus, there was a total of twelve block-separated conditions: Delay 0 ms, 100 ms, 200 ms, 300 ms, 400 ms, 500 ms, 600 ms, 700 ms, 800 ms, 900 ms, 1000 ms, and no visual feedback (“M” condition). Each block was 40 s including the resting and repeated four times in total. The order of the delay conditions was randomized within each run of all the twelve conditions. The subjects took a small break every 24 blocks (two cycles of all the twelve conditions). The flow of the experiment is illustrated in Figure 2. EEG recording was paused during the rest. A total of 32 min of EEG data was recorded from each subject. The experiment was designed and controlled using PsychPy software [12].

During the second part of the experiment, the participants repeated the movement tasks, but, this time, they reported the subjective evaluation of the perceived SoA with an integer scale from 0 to 10 in which the larger the number, the more control on their own movement. Each delay condition was repeated three times randomly in this part, and the “M” condition was omitted. All data displayed on the screen during the whole experiment were recorded using OBS Studio [13], which can be used for further investigation.

### 2.3. Subjective Analysis of the Sense of Agency

The subjective data collected in the second session of the experiment were used to quantitatively assess the changes in the SoA as the amount of delay applied to the visual feedback of the participants own movement changed. The SoA was divided into 11 discrete levels in this experiment for the purpose to let participants estimate the strength of SoA they perceived at each delay condition. The highest level, level 10, matches the zero delay and indicates full SoA, whereas level 0 corresponds to the maximum delay and reflects a lack of agency over their hand movement. Each delay condition was played 3 times and a window pops up on the screen after each condition, and participants should select a number (between 0 and 10) to rate how the displayed movement on the monitor was congruent with their agency over their real hand movement. The recorded rating of the SoA corresponding to each level of delay was averaged over subjects. Mean and standard error of mean (SEM) for data of each condition were computed, and statistical plots were generated.

### 2.4. EEG Data Acquisition and Preprocessing

EEG data were recorded from 16 active EEG electrodes placed on a cap with the locations compatible to international 10–20 system (FP1, F3, F5, F7, C3, T7, P3, P7, O1, O2, P4, P8, C4, T8, F4, F8, Fp2) using V-Amp amplifier (Brain Products GmbH, Gilching, Germany). The ground and the online initial reference electrodes used during EEG recording were placed on Oz and Cz, respectively. The data sampling rate was 2 kHz. Bipolar vertical and horizontal electrooculograms (EOG) were recorded using the auxiliary inputs of the amplifier. No online filters were applied during the data acquisition.

**Figure 3:**
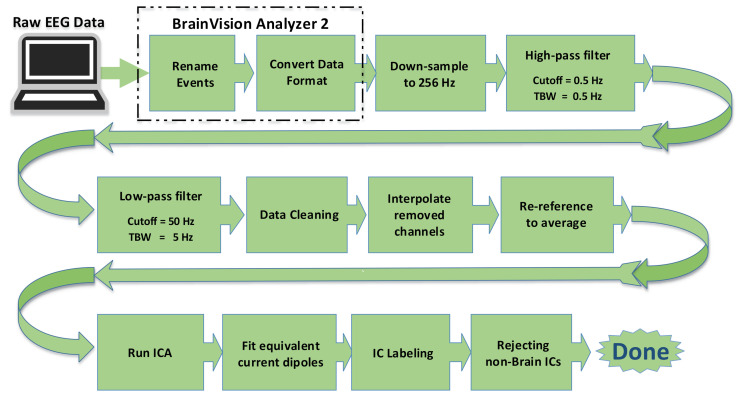
EEG data preprocessing pipeline. The first two steps are done using BrainVision Analyzer 2, while the other steps are performed on MATLAB 2017b.

Offline, the raw EEG data were imported to BrainVision Analyzer 2 [14] and further preprocessed by the EEGLAB toolbox [15] running under MATLAB 2017b (The MathWorks Inc., Natick, MA, USA). The preprocessing steps are shown in the block diagram in Figure 3. The EEG data were first downsampled to 256 Hz. A high-pass filter (with 0.5 Hz low cutoff and transition bandwidth of 0.5 Hz) and a low pass filter (50 Hz high cutoff and 5 Hz transition bandwidth) were applied. We used two-step data cleaning approaches, artifact subspace reconstruction (ASR) [16,17,18,19] and independent component analysis (ICA) [20,21]. These two approaches work in a complementary manner; ASR uses a sliding-window PCA-based subspace rejection and reconstruction allowing it to address data non-stationarity such as infrequent short-lasting bursts, while ICA uses a stationary spatial filter method to find temporally maximally independent sources, which can reveal physiologically valid cortical EEG sources [22], by linearly unmixing the “scalp projection”. EEG data were offline re-referenced to average prior to applying ICA. Subsequently, we fit an equivalent current dipole model to independent component (IC)’s scalp topographies by using Dipfit 3.3 from Fieldtrip [23]. We used EEGLAB’s default electric forward model based on MNI template [24,25] to build a boundary element model (BEM) that consists of three layers of the brain, skull, and scalp, whose electric conductivities are [0.33 0.0041 0.33] S/m, respectively (i.e., brain-to-skull conductivity ratio is 80). The template electrode locations were coregistered to the MNI coordinate system. We used ICLabel [26] to generate seven probabilistic class labels, ’Brain’, ’Muscle’, ’Eye’, ’Heart’, ’Line Noise’, ’Channel Noise’, or ’Other’ for each IC. Finally, all the ’non-Brain’ ICs were rejected. The mean number of ICs rejected was 9.9 (SD 2.5, range 6–16), and the corresponding mean electrode variance reduction was 59% (SD 16, range 32–89). The mean label probability of the ‘Brain’ class was 82% (SD 10, range 55–98).

### 2.5. EEG Data Analysis

For each of the 20-s condition-separated blocks, we calculated the power spectral density (PSD) using EEGLAB function spectopo(). The window size used was 4 s (Hamming window) with 50% overlap, and the frequency bin resolution was 0.25 Hz. The average power of the traditional EEG frequency band was calculated. Table 1 illustrates the EEG frequency bands used in this study. The PSD was computed for both scalp electrode data and IC time series.

To highlight the spatial distribution of the ICs that contribute to the significant results observed at scalp electrode data, spatial probabilistic density of the equivalent current dipoles of the ICs that survived the statistical thresholding and multiple comparisons, described below, were plotted together with their mean values. To compute the dipole density, 3D Gaussian with FWHM of 30 mm was applied to the dipole locations. Then, the group-level sum was calculated for visualization.

### 2.6. Statistical Test

We calculated Pearson’s correlation coefficients and *p*-values. The independent variable was the visual delay (0–1000 ms for every 100 ms). The dependent variable was the average EEG power within the defined frequency band (Delta, Theta, Alpha, Beta1, Beta2, Gamma). In the preliminary analysis, we performed one-way ANOVA for the eleven levels of the visual delay and obtained similar results. However, we decided to use Pearson’s correlation test for the final results because the meaning of the correlation coefficient makes intuitive sense compared with the F-statistics generated by ANOVA. To control the family-wise error rate, multiple comparison correction using a false discovery rate (FDR) [27] was applied. The test results showed general tendency for saturation, so we applied a stringent correction threshold of 18 (electrodes) × 6 (frequency bands) × 15 (subjects) for the scalp electrode data and 8 (average number of ICs) × 6 (frequency bands) × 15 (subjects) for the IC data. This is equivalent to correcting single-subject’s single-electrode/IC for each frequency band, which was necessary and used for the IC preselection but also applied to scalp electrode analysis for the sake of consistency. The results from the scalp electrode analysis showed tendency of saturation, so the risk of the Type II error (i.e., missing the true positive) was unlikely to be a problem. In the same vein, the statistical threshold for the *p*-value was *p* < 0.01 for the scalp electrode data and *p* < 0.05 for the IC data, respectively.

## 3. Results

### 3.1. Behavioral Results

**Figure 4:**
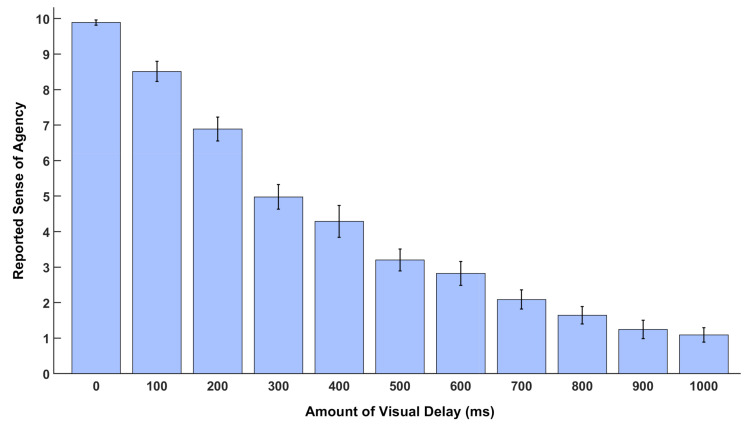
Behavioral results of subjective rating of the Sense of Agency (SoA) with an increasing delay of visual feedback.

The summary of the subjective report of SoA is shown in Figure 4. The group-mean SoA changes are congruent with the amount of the delay in the visual feedback (Pearson’s correlation coefficient, *r* = 0.96, and *p*-value, *p* < 0.001). The behavioral data thus replicated the previous reports [10,11].

### 3.2. EEG Results

**Figure 5:**
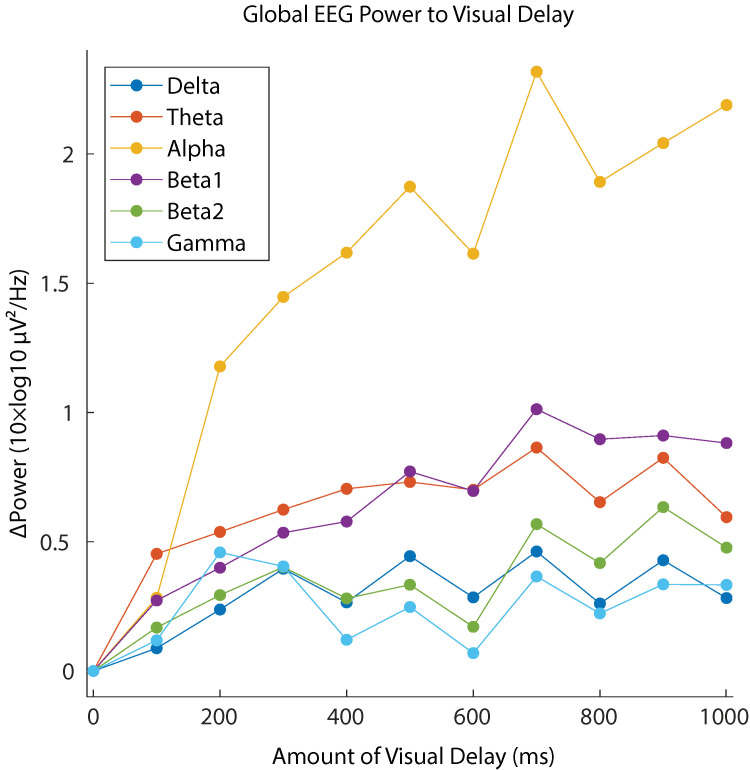
Global EEG power (i.e., mean across all scalp electrodes) changes to increasing amount of visual delay for each frequency band. The changes are relative to no delay condition (0 ms). The alpha-band power showed the most sensitive changes to the increase of visual delay.

Figure 5 shows the grand-mean global (i.e., mean across all the scalp electrodes) EEG power changes for each frequency band. It is clear that alpha band power showed the most sensitive changes to the increased delay of the visual feedback.

**Figure 6:**
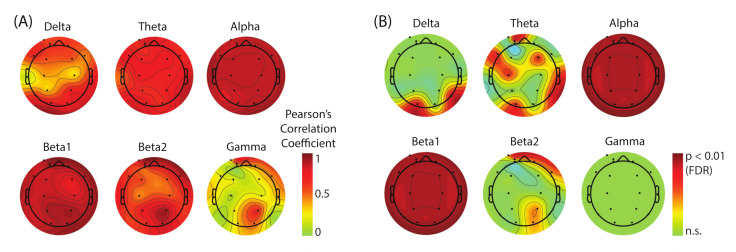
Pearson’s correlation coefficients (**A**) and statistical significance mask with FDR-corrected *p*-value, *p* < 0.01 (**B**). The correlation between the Δpower and the amount of visual delay was tested for all the combination of scalp electrodes and frequency bands, which was corrected using a false discovery rate (18 × 6 × 15 = 1620 tests). Note that alpha and beta1 showed global response to the increase of visual delay. However, the globally saturated result also means poor spatial resolution of the underlying EEG sources.

To study the scalp distribution of the EEG power modulation for each frequency band, we plotted the results from the Pearson’s correlation test in Figure 6. The overall positive values across frequency bands and the scalp locations indicate a global tendency of the EEG power increase together with the increase of the visual feedback delay. The significance masks across the frequency bands indicate that alpha and beta1 are the two sensitive frequency bands. However, the spatial resolution in these frequency bands is poor because of heavy saturation across all the electrodes.

**Figure 7:**
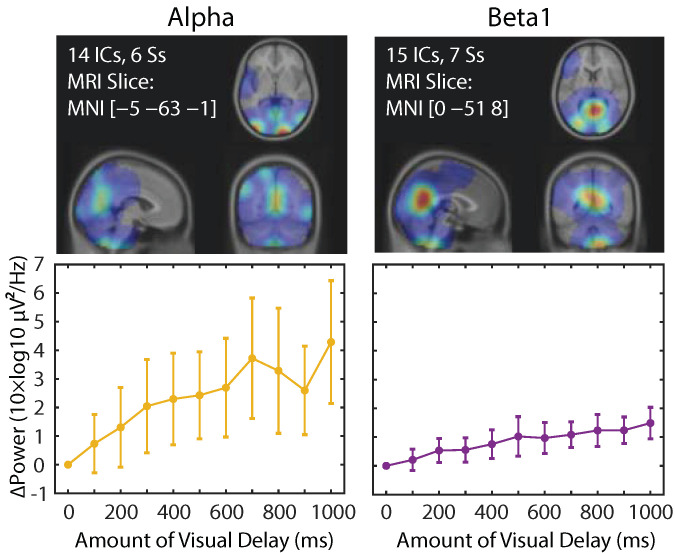
Density of the equivalent current dipoles (Gaussian smoothing with FWHM = 30 mm) fitted to independent components whose power showed significant (FDR-corrected *p*-value, *p* < 0.05 for 10 × 6 × 15 = 900 tests) correlation to the amount of visual delay. Only alpha and beta1 survived the multiple comparison correction, and the estimated locations of the ICs were in the inferior parietal to superior occipital.

We then performed the analysis on single IC, which may be considered as “the effective EEG source” [21,22] (Figure 7). Only alpha and beta1 band power tests survived the multiple comparison correction using FDR. The number of ICs survived were 14 (from six subjects, hereafter Ss) and 15 (7 Ss) for alpha and beta1, respectively. Even after applying the further correction using Benferroni across the six frequency bands, four and five ICs survived for alpha and beta1 frequency bands, respectively. Apparently, the dominant contribution by the alpha and beta1 explains the results at the scalp electrodes. Importantly, the locations of the estimated equivalent current dipoles were mostly parieto-occipital. Thus, we conclude that the dominant source of the delay-related EEG power increase is in the parieto-occipital regions. Note also that the amount of power increase in beta1 is not visibly large compared with alpha frequency band, but still showed consistent tendency. The results suggest a different underlying mechanism between alpha and beta1 frequency bands.

## 4. Discussion

The goal of the present study was to replicate and extend our understanding on the sensitivity of EEG on SoA paradigm using healthy subjects. For this purpose, we searched for the EEG correlate of SoA changes in response to parametrically delayed visual feedback of the hand movement. The subjective results confirmed effectiveness of the paradigm, and the EEG results reflected the subjective changes of SoA. Moreover, the ICA-based spatiotemporal decomposition revealed for the first time the parieto-occipital distribution of the dominant EEG sources in the alpha and lower beta bands. The strength of the current study is that not only does it provide a replication to the limited number of previous studies available [11,28], it also extends our understanding as to where in the brain the sources of the dominant, frequency-specific modulation are distributed.

The subjective report of the SoA confirmed a clear degradation in the perceived SoA as the delay in the visual feedback increased. Similar results have been implicated by several behavioral studies on the SoA [10,11,29]. Correspondingly, the EEG data showed relative spectral power increase with respect to 0 delay in broadband. Our analysis revealed that the alpha band reflected the changes of SoA sensitively across the whole scalp regions (see Figure 6). The lower-beta band (beta1) also showed the strong modulation, but the effect size of the power increase was about half of that in the case of alpha band. We conclude that the alpha band power is the main EEG correlate of the lack of SoA.

We were interested in estimating the spatial distribution of the dominant sources that contribute to this SoA-specific modulation. However, the result showed substantial saturation (FDR-corrected *p*-values, *p* < 0.01) across the whole scalp regions in both alpha and beta1 bands (Figure 6B) which made it unable for us to visually evaluate the location of the underlying sources. To address this issue, we performed ICA-based scalp EEG modeling so that electrode-level EEG is re-constructed as a linear sum of the projections from temporally maximally independent source activities whose locations inside the brain can be estimated as equivalent current dipoles [21,22]. The results from this analysis demonstrated that a greater number of ICs showed a linear power increase as SoA decreased in the alpha and beta1 bands, and their main spatial distribution was found in the parieto-occipital regions (Figure 7). Interestingly, the number of ICs that are dominant contributors to the SoA-related power changes are comparable (14 vs. 15 among all the subjects), but the effect size in EEG power is about two times bigger in the alpha than beta1, replicating the pattern found in the scalp electrode measures. The results may suggest that the alpha band and the beta1 band may represent different processes.

Our findings are in line with several neuroimaging studies on the SoA. It was reported that temporo-parietal junction (TPJ), primary somatosensory cortex, inferior parietal lobe (IPL), parieto-occipital sulcus, and insula [10,30,31,32,33,34] are the neural correlates of the SoA, most of which can be included in the parieto-occipital regions. One of the particularly relevant studies reported that the no-agency status, i.e., the sense of ’It is not my movement’, corresponding to a large time delay in our experiment, is associated with the activation of the parietal regions [33]. Even with the general limitation in the spatial resolution with the 18-chennel time-series data, it still seems valid to conclude that we found the relevant EEG sources within the parieto-occipital areas, at least in the sense that it was not found in the frontal or temporal areas.

Our result is consistent with the results from the previous EEG study [11]. They reported EEG power increase in the alpha and beta bands as the control over hand movement decreased (corresponding to delay increase in our study). Moreover, they reported that these results were found in the central, parietal, and occipital areas, which is also in line with our results. In the current analysis, we further investigated spatial distribution of the effective EEG sources in the sense of ICA. We observed the parieto-occipital distribution of the dominant contributors of the EEG power changes, which provides additional evidence that supports the parieto-occipital involvement in processing the altered visual feedback of self-motion. It is also important to confirm that our results did not observe any dominant frontal source in EEG power analysis, which is also consistent with their results. They concluded that the frontal region is the key brain region to process SoA based on their connectivity analysis, but the reported connectivity seems heavily involved with the fronto-parietal network for the alpha band and the fronto-occipital network for the beta band. Together with the EEG power analysis results, we conclude that the parieto-occipital region is the key region in both power analysis and possibly in the connectivity analysis as well. The current study provides another support for the bridge over the gap between the relatively new paradigm and the rich literature of EEG power analysis.

The current paradigm may also have clinical usefulness. Conventional and virtual reality-based mirror visual feedback (MVF and VR-MVF) systems have been proposed among different methods to cure and release pain in patients with phantom limb, CRPS, and other chronic pain conditions [35,36,37,38]. The effectiveness of MVF therapy systems in alleviating the chronic pain severity is related to the restoration of the closed-loop between the motor command to the affected body part and its anticipated visual feedback [9,37]. This loop is suggested to be involved in the SoA, but the relationship between such therapy and the SoA is still unclear. A subjective study on patients with phantom limb reported improvement in the SoA following a single short session of conventional mirror therapy, suggesting that short-term mirror therapy can enhance the SoA over the phantom limb [9]. In the future studies, EEG experiments on patients with chronic pain conditions may help to better understand the mechanism of MVF/VR-MVF therapy systems in alleviating pain. We believe that the present study provides ground work that clarifies the spatiotemporal regions of interest upon which a clinical target for treatment and monitoring may be built.

One of the limitations of the current study is the minimum number of EEG electrodes used. With more electrodes available, ICA could decompose more brain signals as well as successfully separating the physiological/non-physiological artifacts to improve the overall SNR. In addition, with the greater number of electrodes, the scalp topography of the ICs can be better represented, which generally improves localization of the equivalent current dipole sources as well. We hope that the current study serves as a proof of concept to justify more full-fledged studies using a high-density EEG system in the larger scale of the project with more subjects, such as studying individual differences associated with personality traits for the purpose of individualized medicine.

## 5. Conclusions

We conducted an EEG experiment on healthy subjects to explore the EEG correlates for a sense of agency in response to delayed visual feedback of hand movement. The explicit assessment of the SoA demonstrated a degradation in the perceived SoA as the visual feedback of self-movement was distorted by increasing the amount of time delay applied to the real movement’s visual return. The results revealed that the EEG power in the alpha and low-beta bands linearly increased as the introduced delay to the video feedback increased, and the dominant source of EEG power increase was resolved in the parieto-occipital regions. The results from the current study extend our understanding of the SoA modulation in the context of EEG power analysis.

## Figures and Tables

**Table 1:** EEG frequency bands.

Frequency Band	Range (Hz)
Delta	1–4
Theta	4–8
Alpha	8–13
Early-Beta (Beta1)	13–20
Late-Beta (Beta2)	20–35
Gamma	35–50

## Data Availability

The data analyzed during the current study are available from the corresponding authors on reasonable request.

## Abbreviations

The following abbreviations are used in this manuscript:

CRPS	Complex Regional Pain Syndrome
FDR	False Discovery Rate
fMRI	Functional MRI
IC	Independent Component
ICA	Independent Component Analysis
SoA	Sense of Agency
SoO	Sense of Ownership
VR-MVF	Virtual Reality-based Mirror Visual Feedback
